# Serum and cervicovaginal IgG immune responses against α7 and α9 HPV in non-vaccinated women at risk for cervical cancer: Implication for catch-up prophylactic HPV vaccination

**DOI:** 10.1371/journal.pone.0233084

**Published:** 2020-05-18

**Authors:** Ralph-Sydney Mboumba Bouassa, Hélène Péré, Camélia Gubavu, Thierry Prazuck, Mohammad-Ali Jenabian, David Veyer, Jean-François Meye, Antoine Touzé, Laurent Bélec

**Affiliations:** 1 Laboratoire de virologie, hôpital européen Georges Pompidou, Assistance Publique-Hôpitaux de Paris (AP-HP), Paris, France; 2 Ecole Doctorale Régionale en Infectiologie Tropicale, Franceville, Gabon; 3 Université Paris Descartes, Paris Sorbonne Cité, Paris, France; 4 INSERM UMR_S970, Immunothérapie et traitement anti-angiogénique en cancérologie, Paris Centre de Recherche Cardiovasculaire (PARCC), hôpital européen Georges Pompidou, AP-HP, Paris, France; 5 Service des maladies infectieuses et tropicales, Centre hospitalier régional d’Orléans and Centre Gratuit d’Information, de Dépistage et de Diagnostic (CEGIDD) d’Orléans, Orléans, France; 6 Département des Sciences Biologiques et Centre de Recherche BioMed, Université du Québec à Montréal (UQAM), Montreal, Quebec, Canada; 7 Service de Gynécologie Obstétrique, Centre Hospitalo-Universitaire d’Agondjé et Faculté de Médecine de Libreville, Université des Sciences de la Santé, Libreville, Gabon; 8 UMRINRA ISP 1282, Equipe Biologie des infections à polyomavirus, Université de Tours, Tours, France; Penn State University School of Medicine, UNITED STATES

## Abstract

**Background:**

Cervical cancer associated with high risk-human papillomavirus (HR-HPV) infection is becoming the one of the most common female cancer in many sub-Saharan African countries. First-generation immigrant African women living in Europe are at-risk for cervical cancer, in a context of social vulnerability, with frequent lack of cervical cancer screening and HPV vaccination.

**Objective:**

Our objective was to address immunologically the issue of catch-up prophylactic HPV vaccination in first-generation African immigrant women living in France.

**Methods:**

IgG immune responses and cross-reactivities to α7 (HPV-18, -45 and -68) and α9 (HPV-16, -31, -33, -35, -52 and -58) HPV types, including 7 HR-HPV targeted by the Gardasil-9^®^ prophylactic vaccine, were evaluated in paired serum and cervicovaginal secretions (CVS) by HPV L1-virus-like particles-based ELISA. Genital HPV were detected by multiplex real time PCR (Seegene, Seoul, South Korea).

**Results:**

Fifty-one immigrant women (mean age, 41.7 years; 72.5% HIV-infected) were prospectively included. More than two-third (68.6%) of them carried genital HPV (group I) while 31.4% were negative (group II). The majority (90.2%) exhibited serum IgG to at least one α7/α9 HR-HPV. Serum HPV-specific IgG were more frequently detected in group I than group II (100% *versus* 68.7%; P = 0.002). The distribution of serum and genital HPV-specific IgG was similar, but mean number of IgG reactivities to α7/α9 HR-HPV was higher in serum than CVS (5.6 IgG per woman in serum *versus* 3.2 in CVS; P<0.001). Rates of IgG cross-reactivities against HPV different from detected cervicovaginal HPV were higher in serum and CVS in group I than group II. Finally, the majority of groups I and II women (68.6% and 68.7%, respectively) exhibited serum or cervicovaginal IgG to Gardasil-9^®^ HR-HPV, with higher mean rates in group I than group II (6.1 Gardasil-9^®^ HR-HPV per woman *versus* 1.4; P<0.01). One-third (31.2%) of group II women did not show any serum and genital HPV-specific IgG.

**Conclusions:**

Around two-third of first-generation African immigrant women living in France showed frequent ongoing genital HPV infection and high rates of circulating and genital IgG to α7/α9 HPV, generally cross-reacting, avoiding the possibility of catch-up vaccination. Nevertheless, about one-third of women had no evidence of previous HPV infection, or showed only low levels of genital and circulating HR-HPV-specific IgG and could therefore be eligible for catch-up vaccination.

## Introduction

Human papillomavirus (HPV) infection is the most common viral sexually transmitted infection (STI) worldwide and high risk-HPV (HR-HPV) genotypes, particularly HPV-16 and HPV-18, are responsible for 5.2% of all cancers worldwide and 7.7% of all cancers in developing countries [1–3]. Most genital HR-HPV types cluster in the α7 (HPV-18, -45 and -68) and α9 (HPV-16, -31, -33, -35, -52 and -58) species [4–6].

According to the World Health Organization (WHO), cervical cancer will kill annually about half of a million women by the next decade, mostly in sub-Saharan Africa where cervical cancer is currently the first female cancer in several countries, mainly worsened by the HIV epidemic [7–9]. Thus, cervical cancer has become progressively one of the main public health challenges to overcome in sub-Saharan Africa [10].

The prophylactic vaccination of young girls below 14 years with the safe and very effective Gardasil-9^®^ vaccine (Merck & Co. Inc., Kenilworth, NJ, USA) containing VLPs from HPV-6 and HPV-11, as well as two α7 (HPV-18 and HPV-45) and five α9 (HPV-16, -31, -33, -52 and -58) HR-HPV, constitutes actually one of the main strategies against cervical cancer [10–18]. In addition to the secondary prevention measures, sexually active adult women more than 15 years may also be eligible for catch-up HPV vaccination [10, 19–22].

Most of the first-generation African immigrant women living in Europe has started their sexual life in their home country and could harbor an infectious profile reflecting the epidemiology of their country of origin where cervical HR-HPV infection is highly prevalent [23] and exacerbated by the so-called “syndemic” synergy played by HIV epidemic and other STIs [24]. These women harbor higher genital HR-HPV prevalences compared to the European female population [25,26]. In addition, African immigrant women are subjected to lower HPV vaccine initiation and completion [27–30], and they are less screened for cervical cancer in their lifetime than women born in Europe [31,32].

Taken together, African immigrant adult women living in Europe, particularly those infected with HIV, appear to be at very high risk of developing cervical cancer and the catch-up HPV vaccination in these women constitutes therefore a very promising complementary strategy for the prevention of cervical cancer [20–22]. However, it is still unclear whether catch-up HPV vaccination of immigrant women who are sexually active since a while at time of vaccine introduction would be feasible and beneficial. Presently, the French National Authority for Health (Haute Autorité de Santé, HAS) as many other country in Europe, only recommends the secondary prevention for women from 25 to 65 years without the catch-up HPV vaccination [33,34].

Primary HPV infection induces both CD8+ T cells cytotoxic as well as B cells-derived humoral responses [35]. These two arms of the immune response help to control and clear HPV infection, with the natural B-cells derived antibody response representing the main barrier to prevent new HPV infection [35]. Natural B cells-derived antibody responses against HPV has been described in two mucosal compartments, including the cervicovaginal secretions [36–38] and the saliva [39], as well as in the systemic compartment [40–45]. In the cervicovaginal compartment, HPV-specific IgG are 2.5 times more abundant than HPV-specific IgA and secretory-IgA (s-IgA) [46], as previously demonstrated for HIV infection [47]. These findings point the critical interest to evaluate the HPV-specific immune IgG response in cervicovaginal secretions as a marker of previous or ongoing HPV infections. As for the cervicovaginal mucosa, circulating HPV-specific IgG are more predominant and persistent than the other antibody classes (IgA and IgM) [41, 48–51]. Serum HPV-specific IgG response reflects past-infection with a 50–70% rate of seroconversion in infected women [42,50] or persistent viral infection [48]. Naturally derived HPV-specific IgG antibodies from single-positive sera were highly genotype-specific, while in multi-positive sera, cross-reactive antibodies were observed both within and between some α7 (HPV-18 and -45) and α9 (HPV-16, -31, -33, -52 and -58) strains [45]. The serological IgG immune response to HPV persists over time, it is associated with the cumulative number of lifetime sex partners [50], and it constitutes the “gold standard” serological test for past-history or ongoing HPV infection or reinfection [41,50].

The aim of the study was to address immunologically the issue of catch-up HPV vaccination in first-generation African immigrant women living in France, which constitute a vulnerable and high risk population for HPV infection and cervical cancer. The IgG immune responses against the 7 HR-HPV contained in Gardasil-9^®^ vaccine and cross-reactivities to α7 and α9 HPV types, were evaluated in a prospective series of first-generation immigrant women, in both systemic and genital compartment and in relationship with genital HPV DNA detection, in order to approach the most persisting immune response to HPV during past-infection or ongoing infection and to predict possible efficiency of catch-up prophylactic Gardasil-9^®^ HPV vaccine.

## Material and methods

### Enrolment, selection criteria and study population

**Fig 1:**
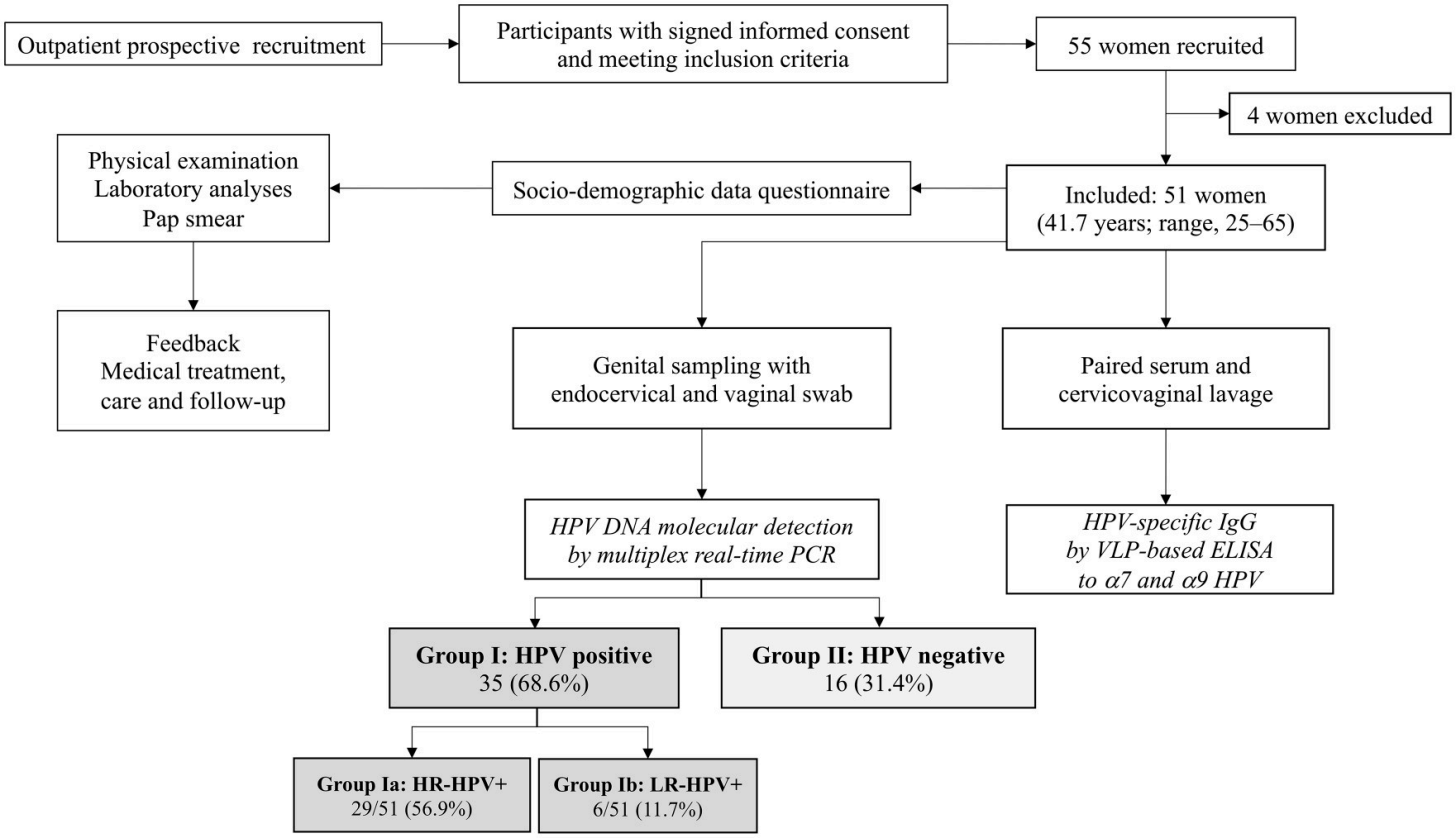
Flow diagram of the *ImmiPap* study. Three biological samples were obtained for study analyses, including a serum sample, a genital swab of endocervix and vaginal walls, and a standardized cervicovaginal lavage with 3 mL of PBS. HPV DNA detection was carried out on nucleic acid extracted from swab-collected genital secretions by multiplex Anyplex^™^ II HPV28 real-time PCR (Seegene, Seoul, Korea). The group I of study women showed HPV DNA in their genital secretions, including HR-HPV DNA with or without associated LR-HPV (group Ia) or exclusively LR-HPV DNA (group Ib); the group II of study women did not show detectable HPV DNA in their genital secretions. The paired serum and acellular part of cervicovaginal lavage samples were analyzed for serum and genital HPV-specific IgG, respectively, using virus-like particles-based indirect ELISA assay against HR-HPV belonging to α7 (HPV-18, -45 and -68) and α9 (HPV-16, -31, -33, -35, -52 and -58) groups. ELISA: Enzyme-linked immunosorbent assay; HR-HPV: High risk-HPV; LR-HPV: Low risk-HPV.

The study population consisted of African immigrant women living in France recruited for the ANRS (*Agence Nationale de Recherches sur le Sida et les hépatites virales*, Paris, France) *ImmiPap* study (Fig 1). The *ImmiPap* study consists in the description of the molecular epidemiology of genital HPV infection in first-generation immigrant adult women coming from HPV endemic countries in sub-Saharan Africa, and living in France, with the ultimate aim to prevent, diagnose and cure cervical cancer. Study women beneficiated of the recently 2019-revised HAS recommendations for the primary molecular screening of genital HPV infection in women aged from 25 to 65 years [34]. The *ImmiPap* study finally focuses on HPV prophylactic vaccination in immigrate women who are at high risk for cervical cancer.

First-generation immigrant African women attending the *Centre Gratuit d’Information*, *de Dépistage et de Diagn*ostic (CeGIDD) of the *Centre Hospitalier Régional d’Orléans* were prospectively included in 2019. The CeGIDD is an outpatient consultation service providing HIV, STIs, hepatitis and HPV prevention, screening and care for the general adult population.

The inclusion criteria were to be a first-generation African immigrant women according to the French National Institute of Statistics and Economic Studies [52], being aged between 25–65 years, being sexually active, having no genital troubles at physical examination, having signed the informed consent form, having fully completed the study questionnaire and providing the blood and genital samples required for the virological and immunological studies. Exclusion criteria included age less than 25 years and more than 65 years, not willing to participate to the study or to answer the face-to-face sociodemographic and medical data questionnaire.

After signed the informed consent form, included study women were invited to fulfill a face-to-face questionnaire that included socio-demographic characteristics and behavioral data such as age, marital status, social occupation, education level, past-history of STI, HIV status and also sexual behavioral characteristics such as the number of lifetime sexual partners, frequency of condom use and the age at first sexual intercourse. Study women were subjected to physical examination with appropriate laboratory analyses when necessary, and to a routine Pap smear cytology, as recommended by the HAS in women aged from 25 to 65 years for HPV-related genital lesions detection [33]. They also benefited from free STIs screening, including HIV, *Chlamydia trachomatis*, genital herpes and syphilis. All women received an information session on HIV, STIs and cervical cancer. Women diagnosed positive for any STIs received adequate care.

### Samples and processing

After completing the questionnaire, a clinician performed genital sampling of the endocervix and the vaginal walls using a flocked swab (Copan Diagnostic Inc., California, USA), frozen at -80°C. Afterwards, whole cervicovaginal secretions (CVS) were collected using a standardized vaginal washing, with 3 mL of phosphate-buffered saline (PBS) (Thermo Fisher Scientific, Waltham, MA, USA), as previously described [53]. The samples, corresponding to an approximately 10-fold dilution the original secretions [53], were centrifuged to remove cells, debris, and insoluble mucus, prior to be stored at -80°C. For each woman, 5 mL of venous blood were obtained in dry tube, and the serum was retrieved to be further aliquoted in 1.5 mL cryotubes before to be stored at -80°C.

Frozen genital and serum samples were transported in frozen ice packs to the virology laboratory of the *hôpital européen Georges Pompidou*, Paris, France, for virological and serological analyses.

Cervical cytology was carried out by cytoscreeners and pathologists blinded to the outcomes of HPV testing. Cytological results were classified according to the Bethesda system 2001 [54].

### HPV detection and genotyping

Genital swab from each included women were subjected to DNA extraction using the DNeasy Blood and Tissue kit (Qiagen, CA, USA) and eluted in 100μL of elution buffer according to the manufacturer instructions. Genital HPV DNA was detected in eluted total DNA from vaginal swab and genotyped using the multiplex real-time PCR assay Anyplex^™^ II HPV28 (Seegene, Seoul, South Korea), as previously described [55].

### Human papillomavirus virus-like particles production

VLPs for nine HPV genotypes (HPV-16, HPV-18, HPV-31, HPV-33, HPV-35, HPV-45, HPV-52, HPV-58, and HPV-68) were produced using biscistronic plasmids vectors (p16sheLL, p18sheLL, p31sheLL, pVITRO-HPV33L1L2, p35sheLL, p45sheLL, p52sheLL, p58sheLL and pVITRO-HPV68L1L2) incorporating each one, a copy of the major L1 and minor L2 capsid protein encoded gene specific for each of the nine HPV genotypes, as described previously [56]. Plasmids p16sheLL, p18sheLL, p45sheLL, p52sheLL and p58sheLL were a gift from John Schiller (Addgene plasmid # 37320; # 37321; # 37322; # 37323; # 46950; # 37324) [57–60]. While plasmid p35sheLL was a gift from Simon Beddows (Addgene plasmid # 40626) [61] and pVITRO-HPV33L1L2 and pVITRO-HPV68L1L2 were gifted by Richard Roden (Addgene plasmid # 52493 and # 52587) [62]. All the plasmids were available in the plasmid repository, Addgene (www.addgene.org).

The production of VLP stocks was performed as previously described [56] using the papillomavirus vectors production protocol (https://ccrod.cancer.gov/confluence/display/LCOTF/PseudovirusProduction) edited by the Laboratory of Cellular Oncology of the Center for Cancer Research (National cancer institute, National Institute of Health, Rockville Pike, Bethesda, Maryland, USA). Briefly, 293 TT cells were transfected with each of the L1/L2 plasmid vectors and incubated during 72 hours at +37°C. Cells were then lysed in a buffer (0.5% Nonidet^™^ P 40 Substitute/1X PBS, Sigma-Aldrich, Saint-Louis, Missouri, USA) before being sonicated 3 times for 15 seconds cycles to break the nuclear membrane and thereby releasing the VLPs. The obtained nuclear extracts were purified by ultracentrifugation at 30,500 rpm (SW 32 Ti Swinging-Bucket rotor, Beckman Coulter, Inc., Brea, CA, USA) for 26 hours at +4°C in a cesium chloride (ClCs) gradient. The fraction containing the VLPs was recovered and diluted in 300μL of Dulbecco’s phosphate-buffered saline (DPBS) (Thermo Fisher Scientific, Waltham, MA, USA) and stored at +4°C before being used for serological analyses. As recommended by Buck and Thompson [56], the Pierce^™^ BCA Protein Assay kit (Thermo Fisher Scientific, Waltham, MA, USA) containing different bovine serum albumin (BSA) standards was used to estimate the amount of the VLPs produced from each of the 9 HPV genotypes. The amount of each VLP in nanogram per milliliter (ng/mL) was deducted from the BSA standard curve at 562 nm. Anti-HPV16-L1 antibody [CamVir 1, ab69] (Abcam, Cambridge, UK) was used as quality control to ensure the specificity of VLP-16 and -31, as well as the non-cross-reactivity of the other VLPs with antibody specific for HPV-16 and phylogenetically close genotypes. Likewise, the anti-HPV18-L1 antibody [abx110595] (Abbexa Ltd, Cambridge, UK) was used as quality control for VLP-18. Finally, the anti-HSV-1/HSV-2 (herpes simplex virus type 1/2 glycoprotein B) antibody (ABIN457436, Antibodies-online, GmbH, Aachen, Germany) was used to ensure the non-cross-reactivity with other virus species.

### VLP-based IgG ELISA

An “in-house” VLP-based indirect enzyme linked immunosorbent assay (ELISA) was carried out on paired serum and acellular part of vaginal lavage of each included woman. Briefly, a 96 well ELISA microtiter plates (NUNC F96 MaxiSorp^™^, Thermo Fisher Scientific, Waltham, MA, USA) were coated with 100μL of PBS containing 200 ng of each purified VLP (VLP-16, VLP-18, VLP-31, VLP-33, VLP-35, VLP-45, VLP-52, VLP-58, and VLP-68) and incubated overnight at +4°C. Each well was blocked with 200 μL of PBS containing 1% of fetal bovine serum (Dominique Dutscher SAS, Brumath, France) during 1 hour at room temperature and vigorously decanted to remove the exceeding and non-coated antigens. The paired serum and genital lavages from each woman were then diluted (1:10 and 1:100 for genital lavage and serum, respectively) in PBS and charged in the corresponding wells for an incubation period of 1 hour in the dark, at +37°C. After each incubation step, the plate was washed 4 times with PBS containing 0.1% of Tween^®^ 20 (Sigma-Aldrich, Saint-Louis, Missouri, USA). Then, the plate was incubated at +37°C, in the dark, during 1 hour with an horseradish peroxidase (HRP)-conjugated mouse anti-human IgG monoclonal antibody [ab7499] (Abcam, Cambridge, UK) diluted in PBS (1:6000, as recommended by the manufacturer) in order to detect serum and genital IgG specific for each VLP. Following incubation and washing steps, 100 μL of enzyme substrate [hydrogen peroxide (H_2_O_2_) Sigma-Aldrich, Saint-Louis, Missouri, USA] and chromogen (ortho-phenylenediamine, Sigma-Aldrich) were added in wells and the plates were placed in the dark for a final incubation period of 30 minutes to allow color development. Reactions were stopped with 0.1 M of sulfuric acid (H_2_SO_4_, Thermo Fisher Scientific, Waltham, MA, USA), and optical density (OD) was read between 450 and 620 nm on a microplate reader (ETIMAX 3000, DiaSorin, Saluggia, Italy). All samples were tested in triplicate for each VLP type.

Cutoff positivity values to define HPV-specific IgG seropositivity were calculated independently for each HPV type by analyzing the mean OD values obtained from children’s sera according to the cutoff algorithm recommended by the global HPV LabNet (mean OD value of a negative control serum panel plus 3 SD) [63,64].

A panel of sera from 20 French children of blinded origins and less than 5 years old was used as negative control for serological assays. In addition, a pool of sera from individuals having received the three doses of Gardasil-9^®^ vaccine (Merck & Co. Inc., NJ, USA) constituted the positive control in serological assays for each of the seven HR-HPV types targeted by the Gardasil-9^®^ vaccine (VLPs from HPV-6, -11, -16, -18, -31, -33, -45, -52 and -58).

### Production of human papillomavirus pseudovirions

The production of HPV L1&L2 based-pseudovirions (PsV) was adapted from to the papillomavirus vectors production protocol of the Laboratory of Cellular Oncology of the Center for Cancer Research, as described above for VPLs production and with slight modifications. The biscistronic plasmids vectors p16sheLL, p18sheLL, p31sheLL, pVITRO-HPV33L1L2, p45sheLL and pVITRO-HPV68L1L2 were used for the construction of HPV-16, -18, -31, -33, -45 and -68-PsVs, respectively. Furthermore, each PsV incorporates a reporter plasmid encoding the luciferase (Promega^™^ pGL4.10[luc2] vector, Promega, Madison, Wisconsin, USA) [61].

In brief, 293TT cells (2 x 10^6^ cells) were grown in Dulbecco′s Modified Eagle′s Medium (DMEM) (Thermo Fisher Scientific) supplemented with 10% FBS, 100 IU/ml penicillin, and 100 μg/ml streptomycin (Gibco, USA) and 50 μg/mL of Fungizone^™^ Amphotericin B (Thermo Fisher Scientific) in F75 flask (75 cm^2^) until the confluency of the cells reached 40–50%. Thereafter, 17 μg of each of the L1/L2 plasmid vectors and 17 μg of the reporter plasmid pGL4.10[luc2] were added to 100 μL of Polyethylenimine (PEI) (Sigma-Aldrich) and 4.9 mL of non-supplemented DMEM (Thermo Fisher Scientific) and kept at room temperature for up to 30 minutes to allow transfection complex formation. The growth medium was completely removed from the F75, 293TT cells were gently washed with DPBS and the plasmid/PEI mixture was then added drop wise to the cells layer and the F75 was placed at +37 °C in a CO_2_ incubator. After 4 hours, 10 mL of supplemented DMEM were added to the cells which were then put into the CO_2_ incubator for 8 hours at +37°C. The growth medium was replaced with fresh medium and the cells were further incubated for 60 hours to allow high yield of pseudovirions production. After a total transfection duration of 72 hours, the 293TT-transfected cells were harvested, washed and pelleted by centrifugation (5000x*g* for 10 minutes at +4°C). The cells pellet was then added in a lysis buffer (0.5% Nonidet^™^ P 40 Substitute/1X PBS, Sigma-Aldrich) before being sonicated 3 times for 15 seconds cycles to break the nuclear membrane and thereby releasing free PsVs in the buffer. The nuclear extract for each PsV was incubated during 24 hours in 5% CO_2_ at +37°C for pseudovirion maturation before to be chilled on ice for 5 minutes and clarified by centrifugation (5000x*g* for 10 minutes at +4°C). The solution containing mature PsVs was aliquoted, annotated and stored at -70°C before further analyses.

To evaluate the infectivity of the pseudovirions, the linear expression of the reporter gene and to perform the titration of pseudovirions stocks, COS-7 cells were grown (10^4^ cells/well) in supplemented DMEM in 96-well plates (Thermo Fisher Scientific) in 5% CO_2_ at +37°C until cell confluency was 40–50%. The cells were transduced by replacing the culture medium with fresh non-supplemented DMEM containing serial five-fold dilutions of the previously produced pseudovirions stock solutions and incubated at +37°C. A well containing 1 μg of pGL4.10[luc2] was constituted as a positive control and another one, the cell control for background luminescence (no PsV or reporter plasmid) was also constituted. After an incubation period of 3 hours, 100 μL of growth medium was added to each well and transduced COS-7 cells were incubated during 48 hours at +37°C in 5% CO_2_. Thereafter, the growth medium was removed and transduced COS-7 cells were washed with DPBS and incubated in the dark, during 15 minutes in 100 μL of Pierce^™^ Firefly Luciferase One-Step Glow Assay solution (Thermo Fisher Scientific). Cells lysates obtained were harvested and transferred to a LumiNuncTM 96-well white microplates (Thermo Fisher Scientific) and the luciferase enzyme activity was measured using the Luminoskan ascent (Thermo Fisher Scientific) after adding 50 μL of luciferin substrate to each well. Pseudovirions dilution that yielded at least 80% of the positive control luminescence, after adjusted with background luminescence were selected as suitable and therefore selected as the working dilution for neutralization experiments.

### HPV L1&L2-based pseudovirions neutralization assay

A subsets of four paired sera and cervicovaginal secretions lavage samples were randomly selected from study whose serum and genital samples were of sufficient quantity to undergone pseudovirion-based neutralization assay (PBNA).

For PBNA, total IgG were purified from paired serum and cervicovaginal secretions lavage samples from each selected woman. Thus, for each woman, 6 samples were obtained [original serum and CVS, purified serum IgG (serum IgG), purified genital IgG (genital IgG), serum and CVS depleted of IgG]. The IgG purification from biological paired sera and CVS was performed using the Protein A Splin Plates for IgG Screening kit (Thermo Fisher Scientific) which give high yield of purified IgG, as assessed by further validation using the Pierce^™^ BCA Protein Assay (Thermo Fisher Scientific) (not shown).

The original serum and CVS, as well as purified serum IgG, purified genital IgG, and serum and CVS depleted of IgG, were tested for neutralizing antibodies detection. Briefly, COS-7 cells were pre-plated (10^4^ cells/well) in supplemented DMEM in 96-well plates and incubated 24 hours in 5% CO_2_ at +37°C. Each sample and corresponding PsV working solution were both diluted in the same tube, in non-supplemented DMEM, to reach their corresponding working dilution factor. The mixture was gently homogenized and kept at room temperature for 10 minutes and 50 μL of the mixture were added dropwise to each well after removing the growth medium from the COS-7 layer. To avoid non-specific reactivity, the final dilution of each original and derived samples were 1:100. Infected cells were grown overnight at +37°C and then fed with 100 μL of supplemented DMEM. After an additional 24 hours of growth at +37°C, the medium was removed and cells were washed with DPBS and incubated in the dark, during 15 minutes in 100 μL of Pierce^™^ Firefly Luciferase One-Step Glow Assay solution (Thermo Fisher Scientific). Cells lysates were harvested and transferred to a LumiNunc^™^ 96-well white microplates and the luciferase enzyme activity was measured as described above. A negative control well (no inhibition of the luminescent signal) containing 1 μg of pGL4.10[luc2] and a well containing only COS-7 cells (no PsV or reporter plasmid) for the background luminescence control, were constituted. The inhibition of the luminescence signal >80% was considered as the effective neutralization of the PsV transduction of the COS-7 cells. A pool of sera from individuals having received the three doses of Gardasil-9^®^ vaccine (Merck & Co. Inc.) constituted the positive control for the neutralization assays (luminescence signal inhibition of >80%). All experiments were performed in triplicate.

### Statistical analysis

Means and standard deviations (SD) were calculated for quantitative variables and proportions for qualitative variables. Mann-Whitney test was used to compare mean seroreactivity for each HPV type between women. Wilcoxon signed ranks test (quantitative variables) and McNemar’s Chi-squared test (qualitative variables) were used to compare the mean IgG reactivities and the prevalences of each HPV-specific IgG between paired serum and cervicovaginal samples.

### Ethic statement

This study was part of the *ARNS-ImmiPap* study and the Scientific Committee of the *Centre Hospitalier Régional d’Orléans* formally approved the study. All the included women gave their informed signed consent to participate to the study. In addition, vaccinated individuals who provided serum as positive controls also gave their informed signed consent allowing the use of their blood for the biological analyses of this study. Finally, we also obtained from parents of the 20 children whose sera were used as negative controls, an informed signed authorization allowing the use of their children’s blood for the biological analyses of this study.

## Results

### Sociodemographic and clinical characteristics of study population

A total of 55 African first-generation immigrant women (mean age, 41.7 years; range, 25–65) living in France for an average of 10.7 years (range, 1–32 years) was prospectively recruited (Table 1). Four of them were excluded because of menstruations at time of enrollment. Finally, 51 women fulfilling the inclusion criteria were included. The group I of study women (68.6%, 35/51) showed HPV DNA in their genital secretions, including HR-HPV DNA associated, or not, with low risk-HPV (LR-HPV) (group Ia) or exclusively LR-HPV DNA (group Ib); the group II of study women (31.4%, 16/51) did not show detectable HPV DNA in their genital secretions (Table 1).

**Table 1:** Sociodemographic characteristics, HIV serostatus, past-history of sexually transmitted infections and cervical cytological results in study women, according the presence or absence of HPV DNA detected by multiplex real-time PCR in swab-collected cervicovaginal secretions.

	All study women (N = 51)	Women with genital HPV DNA (group I; N = 35)	Women without genital HPV DNA (group II; N = 16)
**Age** [mean, (range); years]	41.7 (25–65)	42.8 (28–65)	39.6 (24–50)
**Time of stay in France** [mean, (range); years]	10.7 (1–32)	9.8 (1–32)	11.43 (1–27)
**Marital status**			
**Single** [n,(%)]	23 (45.1%)	13 (37.2%)	10 (62.5%)
**In couple** [n,(%)]	20 (39.3%)	15 (42.8%)	5 (31.2%)
**Divorced** [n,(%)]	7 (13.7%)	6 (17.2%)	1 (6.3%)
**Widowed** [n,(%)]	1 (1.9%)	1 (2.8%)	0 (0%)
**Employment status**			
**Employed** [n,(%)]	19 (37.2%)	11 (31.4%)	8 (50.0%)
**Unemployed** [n,(%)]	29 (56.8%)	22 (62.9%)	7 (47.7%)
**Student** [n,(%)]	3 (5.9%)	2 (5.7%)	1 (6.3%)
**Sexual behavior**			
**Age at first sexual intercourse** [mean, (range); years]	18.1 (12–25)	18.1 (12–25)	18.1 (14–22)
**Number of sexual partners in the last year** [mean, (range)]	1.1 (1–2)	1.1 (1–2)	1.1 (1–2)
**Condom use**			
**Always** [n,(%)]	6 (11.7%)	5 (14.3%)	1 (6.3%)
**Occasionally** [n,(%)]	30 (58.9%)	19 (54.3%)	11 (68.7%)
**Never** [n,(%)]	15 (29.4%)	11 (31.4%)	4 (25.0%)
**Seropositivity for HIV infection** [n,(%)]	38 (74.5%)	27 (77.1%)	11 (68.7%)
Past-history of sexually transmitted infections* [n,(%)]	3 (5.8%)	1 (2.8%)	2 (12.5%)
**Cervical cytological results**			
**Normal cytology** [n,(%)]	43 (84.4%)	28 (80.0%)	15 (93.7%)
**LSIL** [n,(%)]	6 (11.7%)	5 (14.3%)	1 (6.3%)
**HSIL** [n,(%)]	2 (3.9%)	2 (5.7%)	0 (0%)

*Sexually transmitted infections included *Chlamydia trachomatis*, herpes simplex virus-type 2 and syphilis.

HSIL: High grade squamous intraepithelial lesion; LSIL: Low grade squamous intraepithelial lesion.

The study population was mainly constituted by HIV-infected women (72.5%; 37/51) while 27.5% were HIV-negative. Single women (45.1%) were more represented than women living in couple with a male partner (39.3%). Only one-third (37.2%) of women reported to have a paid job while more than half of them (56.9%) were unemployed, including a minority (5.9%) of students. None of the students included were infected with HIV (Table 2). The majority of women started their sexual life at 18.1 years (range, 12–25), and all, but 3, reported having started their sexual life in their home country, longtime before moving to France. Most women reported having had an average of 1.1 sexual partners (range, 1–2) during the last 12 months. Most women used generally condom occasionally (58.9%); nearly one-third (29.4%) of study women never used condom; and only a minority used condom (11.7%) consistently. HIV-infected women reported more frequently irregular use or non-use of condom than uninfected women (P < 0.05) (Table 2). The large majority of study women were free of STIs (94.1%), while only a minority (5.9%) reported past-history of STIs. Finally, most women showed normal cytological results (84.4%) and only 8 (15.7%) women exhibited abnormal cytology including 6 women with low-grade squamous intraepithelial lesion (LSIL), and 2 with high-grade squamous intraepithelial lesion (HSIL). Women shedding genital HPV DNA showed a trend to harbor more frequently abnormal cytology results (7 cases, 20.0%) than women without genital HPV DNA (1 case, 6.3%), but the difference was not significant. Interestingly, both cases of HSIL were observed in the group I of women carrying genital HPV DNA.

**Table 2:** Characteristics of the study women according to their HIV serostatus.

	HIV-positive women (N = 38)	HIV-negative women (N = 13)	P**
**Age** [mean, (range); years]	41.7 (25–65)	42.8 (28–65)	NS
**Time of stay in France** [mean, (range); years]	10.7 (1–32)	9.8 (1–32)	NS
**Marital status**			
**Single** [n,(%)]	14 (36.8%)	9 (69.2%)	0.057
**In couple** [n,(%)]	17 (44.7%)	3 (23.1%)	NS
**Divorced** [n,(%)]	6 (15.8%)	1 (7.7%)	NS
**Widowed** [n,(%)]	1 (2.7%)	0 (0.0%)	NS
**Employment status**			
**Employed** [n,(%)]	17 (44.7%)	2 (15.4%)	0.09
**Unemployed** [n,(%)]	21 (55.3%)	8 (61.5%)	NS
**Student** [n,(%)]	0 (0.0%)	3 (23.1%)	0.013
**Sexual behavior**			
**Age at first sexual intercourse** [mean, (range); years]	18.1 (12–25)	18.1 (12–25)	
**Number of sexual partners in the last year** [mean, (range)]	1.1 (1–2)	1.1 (1–2)	
**Condom use**			
**Always** [n,(%)]	6 (15.8%)	0 (0.0%)	NS
**Occasionally** [n,(%)]	17 (44.7%)	13 (100.0%)	0.00027
**Never** [n,(%)]	15 (39.5%)	0 (0.0%)	0.0055
Past-history of sexually transmitted infections* [n,(%)]	2 (5.3%)	1 (7.7%)	NS
**Cervical cytological results**			
**Normal cytology** [n,(%)]	31 (84.4%)	12 (92.3%)	NS
**LSIL** [n,(%)]	5 (11.7%)	1 (7.7%)	NS
**HSIL** [n,(%)]	2 (3.9%)	0 (0.0%)	NS
**Genital HPV infection** [n,(%)]			
**HPV DNA**	27 (71.1%)	8 (61.5%)	NS
**HR-HPV**	24 (63.1%)	5 (38.4%)	NS
**Multiple HR-HPV**	6 (15.8%)	2 (15.4%)	NS
**HPV-specific IgG response in systemic and cervicovaginal compartments** [n,(%)]			
**Systemic compartment**			
**HR-HPV-specific IgG reactivity**	34 (89.5%)	12 (92.3%)	NS
**Multiple HR-HPV-specific IgG reactivity**	30 (78.9%)	9 (69.2%)	NS
**IgG reactivity against at least 3 HR-HPV types**	26 (68.4%)	7 (53.8%)	NS
**IgG reactivity against at least 6 HR-HPV types**	22 (57.9%)	7 (53.8%)	NS
**IgG reactivity against all 9 HR-HPV types**	17 (44.7%)	5 (38.5%)	NS
**Cervicovaginal compartment**			
**HR-HPV-specific IgG reactivity**	33 (86.8%)	10 (76.9%)	NS
**Multiple HR-HPV-specific IgG reactivity**	24 (63.1%)	5 (38.5%)	NS
**IgG reactivity against at least 3 HR-HPV types**	20 (52.6%)	4 (30.7%)	NS
**IgG reactivity against at least 6 HR-HPV types**	12 (31.6%)	0 (0.0%)	0.02
**IgG reactivity against all 9 HR-HPV types**	2 (3.9%)	0 (0.0%)	NS

*Sexually transmitted infections included *Chlamydia trachomatis*, herpes simplex virus-type 2 and syphilis.

**P values were calculated using Pearson χ2 or Fisher exact tests for qualitative variables and Non-parametric Mann-Whitney test for quantitative variables.

HIV: Human Immunodeficiency Virus; HSIL: High grade squamous intraepithelial lesion; HPV: Human Papillomavirus; HR-HPV: High-Risk Human Papillomavirus; IgG: Immunoglobulin G; LSIL: Low grade squamous intraepithelial lesion.

There was no significant difference between the groups I and II for sociodemographic as well as clinical findings (not shown).

No study women were vaccinated against HPV infection and all of them had never undergone cervical Pap smear cytology before the study inclusion. Furthermore, all study women reported to have never been screened for primary molecular detection of HPV DNA in their genital secretions.

### HPV DNA detection and genotyping

The Table 3 summarizes the distribution of HPV genotypes in study women. More than two-third (68.6%, 35/51) of women carried genital HPV DNA (group I), including 56.8% (29/51) carrying genital HR-HPV DNA (group Ia) and a minority (11.7%, 6/51) genital LR-HPV DNA (group Ib). Nearly one-third (31.4%; 16/51) did not show by molecular biology any HPV DNA in their genital secretions (group II). Genital HPV infection profiles with multiple HPV genotypes were observed in around one-quarter of study women (23.5%), with an average of 2.1 HR-HPV (range, 1 to 4) per genital swab sample. The α7 HR-HPV-68 was the most frequently (19.6%) detected genotype, followed by the α9 HR-HPV-58 (13.7%). HR-HPV-16 and HR-HPV-18 were respectively detected in 7.8% and 9.8% of study women. Apart from the Gardasil-9^®^ vaccine HPV-31 type detected in 7.8% of women, each of the other Gardasil-9^®^ types (HPV-6, HPV-11, HPV-33, HPV-45 and HPV-52) were detected only in one (1.9%) woman. Except for the HPV-51 (7.8%), the other HR-HPV types not targeted by the Gardasil-9^®^ vaccine (HPV-35, HPV-39, HPV-56 and HPV-59) were rarely observed.

**Table 3:** HPV-specific IgG immune response in systemic and cervicovaginal compartments, correspondence between HPV types in genital secretions and serum or cervicovaginal IgG reactivities and cross-reactivity of serum or cervicovaginal HPV-specific IgG against HPV types different from detected cervicovaginal DNA HPV, among study women with positive HPV DNA detection in their cervicovaginal secretions (group I; N = 35), including women with HR-HPV DNA (group Ia; N = 29) and women with LR-HPV DNA (group Ib; N = 6), and among women with negative HPV DNA detection in their cervicovaginal secretions (group II; N = 16). The grey cases in group Ia correspond to concordance between HR-HPV DNA types and serum or cervicovaginal HPV-specific IgG.

				HPV-specific immune response																					
		ID	HPV detection*	Systemic compartment									Cervicovaginal compartment												
				α7 HPV**			α9 HPV**						α7 HPV			α9 HPV						Concordance^§^		Cross-reactivity^$^	
				18^μ^	45^μ^	68	16^μ^	31^μ^	33 ^μ^	35	52 ^μ^	58^μ^	18	45	68	16	31	33	35	52	58	Se	CVS	Se	CVS
^**Group I: Positive cervicovaginal HPV DNA**^	^**Group Ia [HR-HPV]**^	**#03**	58	**+**	**+**	**+**	**+**	**+**	**+**	**+**	**+**	**+**	**+**	**+**	**-**	**+**	**+**	**-**	**+**	**+**	**+**	1/1	1/1	8	6
		**#04**	18	**+**	**+**	**+**	**+**	**+**	**+**	**+**	**+**	**+**	**+**	**+**	**+**	**+**	**+**	**-**	**+**	**+**	**+**	1/1	1/1	8	7
		**#05**	59	**-**	**-**	**-**	**+**	**+**	**-**	**-**	**-**	**-**	**+**	**-**	**+**	**+**	**+**	**-**	**-**	**-**	**-**	NA	NA	2	4
		**#06**	18	**+**	**+**	**+**	**+**	**+**	**+**	**+**	**+**	**+**	**+**	**+**	**+**	**+**	**+**	**+**	**+**	**+**	**+**	1/1	1/1	8	8
		**#09**	58	**+**	**-**	**-**	**+**	**+**	**-**	**-**	**+**	**+**	**+**	**+**	**+**	**+**	**+**	**-**	**-**	**+**	**+**	1/1	1/1	3	6
		**#10**	56	**-**	**+**	**+**	**+**	**+**	**-**	**+**	**+**	**+**	**-**	**-**	**-**	**+**	**-**	**-**	**-**	**-**	**-**	NA	NA	7	1
		**#13**	58, 68	**+**	**+**	**+**	**+**	**+**	**+**	**+**	**+**	**+**	**+**	**+**	**+**	**+**	**+**	**-**	**+**	**+**	**+**	2/2	2/2	7	6
		**#14**	58	**+**	**+**	**+**	**+**	**+**	**+**	**+**	**+**	**+**	**+**	**+**	**-**	**-**	**+**	**-**	**+**	**+**	**+**	1/1	1/1	8	5
		**#15**	33	**+**	**+**	**+**	**+**	**+**	**+**	**+**	**+**	**+**	**-**	**-**	**-**	**-**	**-**	**-**	**-**	**+**	**-**	1/1	0/1	8	1
		**#16**	31	**+**	**+**	**+**	**+**	**+**	**+**	**+**	**+**	**+**	**-**	**-**	**-**	**-**	**-**	**-**	**-**	**-**	**-**	1/1	0/1	8	0
		**#25**	58	**+**	**+**	**+**	**+**	**+**	**+**	**+**	**+**	**+**	**+**	**+**	**-**	**+**	**+**	**-**	**+**	**+**	**+**	1/1	1/1	8	6
		**#26**	68	**-**	**+**	**-**	**-**	**+**	**-**	**-**	**+**	**+**	**-**	**-**	**-**	**+**	**+**	**-**	**-**	**-**	**-**	0/1	0/1	4	2
		**#27**	58	**+**	**+**	**+**	**+**	**+**	**+**	**+**	**+**	**+**	**-**	**+**	**-**	**-**	**-**	**-**	**+**	**+**	**-**	1/1	0/1	8	3
		**#29**	39, 51, 58, 68	**+**	**+**	**+**	**+**	**+**	**+**	**+**	**+**	**+**	**-**	**+**	**-**	**-**	**+**	**-**	**+**	**+**	**+**	2/2	1/2	7	4
		**#30**	16, 51, 68	**+**	**+**	**+**	**+**	**+**	**+**	**+**	**+**	**+**	**-**	**-**	**-**	**+**		**-**	**-**	**-**	**-**	2/2	1/2	7	0
		**#31**	68	**+**	**-**	**+**	**+**	**+**	**-**	**-**	**+**	**-**	**+**	**+**	**+**	**+**	**+**	**-**	**-**	**+**	**+**	1/1	1/1	4	6
		**#33**	18, 51	**+**	**+**	**+**	**+**	**+**	**+**	**+**	**+**	**+**	**+**	**+**	**-**	**+**	**+**	**-**	**+**	**+**	**+**	1/1	1/1	8	6
		**#35**	18	**+**	**+**	**+**	**+**	**+**	**+**	**+**	**+**	**+**	**-**	**+**	**-**	**-**	**-**	**-**	**+**	**+**	**+**	1/1	0/1	8	4
		**#37**	68	**+**	**+**	**+**	**+**	**+**	**+**	**+**	**+**	**+**	**-**	**+**	**-**	**-**	**+**	**-**	**+**	**+**	**+**	1/1	0/1	8	5
		**#39**	68	**+**	**+**	**+**	**+**	**+**	**+**	**+**	**+**	**+**	**-**	**+**	**-**	**-**	**-**	**-**	**+**	**+**	**+**	1/1	0/1	8	4
		**#40**	51, 52, 68	**+**	**+**	**+**	**+**	**+**	**+**	**+**	**+**	**+**	**-**	**-**	**-**	**-**	**-**	**-**	**-**	**-**	**-**	2/2	0/2	7	0
		**#42**	68	**+**	**+**	**+**	**+**	**+**	**+**	**+**	**+**	**+**	**-**	**+**	**-**	**-**	**+**	**-**	**+**	**+**	**+**	1/1	0/1	8	5
		**#43**	68	**-**	**-**	**+**	**+**	**+**	**-**	**-**	**+**	**+**	**-**	**-**	**-**	**+**	**-**	**-**	**-**	**+**	**+**	1/1	0/1	4	3
		**#44**	16, 31, 56	**+**	**+**	**+**	**+**	**+**	**+**	**+**	**+**	**+**	**-**	**-**	**-**	**+**	**+**	**-**	**+**	**+**	**+**	2/2	2/2	7	3
		**#46**	45	**+**	**+**	**+**	**+**	**+**	**+**	**+**	**+**	**+**	**-**	**-**	**-**	**-**	**-**	**-**	**-**	**+**	**+**	1/1	0/1	8	2
		**#47**	31	**+**	**+**	**+**	**+**	**+**	**+**	**+**	**+**	**+**	**+**	**+**	**-**	**+**	**+**	**-**	**+**	**+**	**+**	1/1	1/1	8	6
		**#48**	16, 18, 31	**+**	**+**	**+**	**+**	**+**	**+**	**+**	**+**	**+**	**-**	**+**	**-**	**-**	**-**	**-**	**+**	**+**	**+**	3/3	0/3	6	4
		**#50**	35	**+**	**+**	**-**	**+**	**+**	**+**	**+**	**+**	**+**	**-**	**-**	**-**	**-**	**+**	**-**	**+**	**+**	**+**	1/1	1/1	7	3
		**#51**	16	**+**	**+**	**+**	**+**	**+**	**+**	**+**	**+**	**+**	**+**	**+**	**+**	**+**	**+**	**-**	**+**	**+**	**+**	1/1	1/1	8	7
	^**Group Ib [LR-HPV]**^	**#01**	70	**+**	**+**	**+**	**+**	**+**	**-**	**-**	**+**	**-**	**-**	**-**	**-**	**+**	**-**	**-**	**-**	**-**	**-**	NA^£^	NA^£^	6	1
		**#07**	53	**+**	**+**	**-**	**+**	**+**	**-**	**+**	**+**	**+**	**-**	**+**	**-**	**+**	**+**	**-**	**-**	**+**	**-**	NA	NA	7	4
		**#17**	42	**+**	**+**	**+**	**-**	**+**	**-**	**-**	**+**	**+**	**+**	**+**	**+**	**+**	**+**	**+**	**+**	**+**	**+**	NA	NA	6	9
		**#18**	40	**-**	**-**	**-**	**-**	**-**	**-**	**-**	**+**	**-**	**-**	**-**	**-**	**+**	**-**	**-**	**-**	**-**	**-**	NA	NA	1	1
		**#24**	40	**+**	**-**	**+**	**+**	**+**	**-**	**+**	**+**	**+**	**-**	**-**	**-**	**+**	**-**	**-**	**-**	**-**	**+**	NA	NA	7	2
		**#45**	6	**+**	**+**	**+**	**+**	**+**	**-**	**+**	**+**	**+**	**-**	**-**	**-**	**+**	**-**	**-**	**-**	**-**	**-**	NA	NA	8	1
^**Group II: Negative cervicovaginal HPV DNA** (N = 16)^		**#02**	Negative	**-**	**-**	**-**	**-**	**-**	**-**	**-**	**-**	**-**	**-**	**-**	**-**	**-**	**-**	**-**	**-**	**-**	**-**	NA^£^	NA^£^	0	0
		**#08**	Negative	**-**	**-**	**-**	**+**	**+**	**-**	**-**	**-**	**-**	**-**	**-**	**-**	**+**	**+**	**-**	**-**	**-**	**-**	NA	NA	2	2
		**#11**	Negative	**-**	**-**	**-**	**-**	**-**	**-**	**-**	**-**	**-**	**-**	**-**	**-**	**-**	**-**	**-**	**-**	**-**	**-**	NA	NA	0	0
		**#12**	Negative	**-**	**-**	**-**	**-**	**-**	**-**	**-**	**+**	**-**	**-**	**-**	**-**	**-**	**-**	**-**	**-**	**-**	**-**	NA	NA	1	0
		**#19**	Negative	**-**	**-**	**-**	**-**	**-**	**-**	**-**	**-**	**-**	**-**	**-**	**-**	**-**	**-**	**-**	**-**	**-**	**-**	NA	NA	0	0
		**#20**	Negative	**-**	**-**	**-**	**-**	**-**	**-**	**-**	**-**	**-**	**-**	**-**	**-**	**-**	**-**	**-**	**-**	**-**	**-**	NA	NA	0	0
		**#21**	Negative	**-**	**-**	**-**	**-**	**-**	**-**	**+**	**-**	**-**	**-**	**-**	**-**	**-**	**+**	**-**	**-**	**-**	**-**	NA	NA	1	1
		**#22**	Negative	**-**	**-**	**-**	**+**	**-**	**-**	**-**	**-**	**-**	**-**	**-**	**-**	**+**	**+**	**-**	**-**	**-**	**-**	NA	NA	1	2
		**#23**	Negative	**-**	**-**	**-**	**+**	**-**	**-**	**-**	**-**	**-**	**-**	**-**	**-**	**-**	**+**	**-**	**-**	**-**	**-**	NA	NA	1	1
		**#28**	Negative	**-**	**-**	**-**	**-**	**-**	**-**	**-**	**-**	**-**	**-**	**-**	**-**	**-**	**-**	**-**	**-**	**-**	**-**	NA	NA	0	0
		**#32**	Negative	**-**	**-**	**-**	**-**	**+**	**-**	**-**	**-**	**-**	**-**	**-**	**-**	**-**	**+**	**-**	**-**	**-**	**-**	NA	NA	1	1
		**#34**	Negative	**-**	**-**	**-**	**+**	**+**	**-**	**-**	**-**	**-**	**-**	**-**	**-**	**-**	**+**	**-**	**-**	**-**	**-**	NA	NA	2	1
		**#36**	Negative	**-**	**-**	**-**	**+**	**+**	**-**	**-**	**-**	**-**	**-**	**-**	**-**	**-**	**+**	**-**	**-**	**-**	**-**	NA	NA	2	1
		**#38**	Negative	**-**	**-**	**-**	**+**	**+**	**-**	**-**	**-**	**-**	**-**	**-**	**-**	**-**	**+**	**-**	**-**	**-**	**-**	NA	NA	2	1
		**#41**	Negative	**-**	**-**	**-**	**+**	**+**	**-**	**-**	**-**	**-**	**-**	**-**	**-**	**-**	**+**	**-**	**-**	**-**	**-**	NA	NA	2	1
		**#49**	Negative	**-**	**-**	**-**	**-**	**+**	**-**	**-**	**-**	**-**	**-**	**-**	**-**	**-**	**+**	**-**	**-**	**-**	**-**	NA	NA	1	1

* HPV genotypes detected by Anyplex HPV 28 molecular test kit (Seegene, Seoul, Korea): LR-HPV: 6; 11; 40; 42; 43; 44; 53; 54 and 70, HR-HPV: 16; 18; 31; 33; 35; 39; 45; 51; 52; 56; 58; 59 and 68, Possibly oncogenic HPV: 26; 61; 66; 69; 73 and 82;

** α7 high risk-HPV types: HPV-18, -45 and -68; α9 high risk-HPV types: HPV-16, -31, -33, -35, -52 and -58;

^μ^ HPV-16, -18, -31, -33, -45, -52 and -58 are the HR-HPV genotypes targeted by the Gardasil-9^®^ vaccine;

^§^ Concordance is the n/n’ ratio corresponding to the number (n) of cervicovaginal HPV types detected by PCR out of the number (n’) of detectable HPV-specific serum or cervicovaginal IgG positivity against the same HPV types: 1/1, 2/2 or 3/3 correspond to perfect concordance; 0/1, 0/2, 0/3 correspond to lack of concordance and 1/2 correspond to serum or cervicovaginal reactivity against 1 out of the two HPV types detected by PCR;

^$^ Cross-reactivity corresponds to the number of serum or cervicovaginal HPV-specific IgG reactivities against HPV types other than those detected in genital secretions of women from group I, and to the number of serum or cervicovaginal HPV-specific IgG reactivities against any HPV types in women from group II;

^£^ Not applicable for women infected with HPV types other than the 9 HR-HPV types (HPV-16, -18, -31, -33, -35, -45, -52, -58 and -68) used to produce the virus-like particles constituting the HPV antigens in the IgG ELISA, or for women not infected by HPV in their genital tract.

CVS: Cervicovaginal secretions; ELISA: Enzyme-linked immunosorbent assay; HPV: Human papillomavirus; HR-HPV: High risk-HPV; ID: Identification number; IgG: Immunoglobulin G; Low risk-HPV: LR-HPV; NA: Not applicable; Se: Serum.

### Serum HPV-specific IgG immune response

The Table 3 summarizes serum and vaginal humoral IgG immune responses against α7 and α9 HR-HPV types in study women.

**Fig 2:**
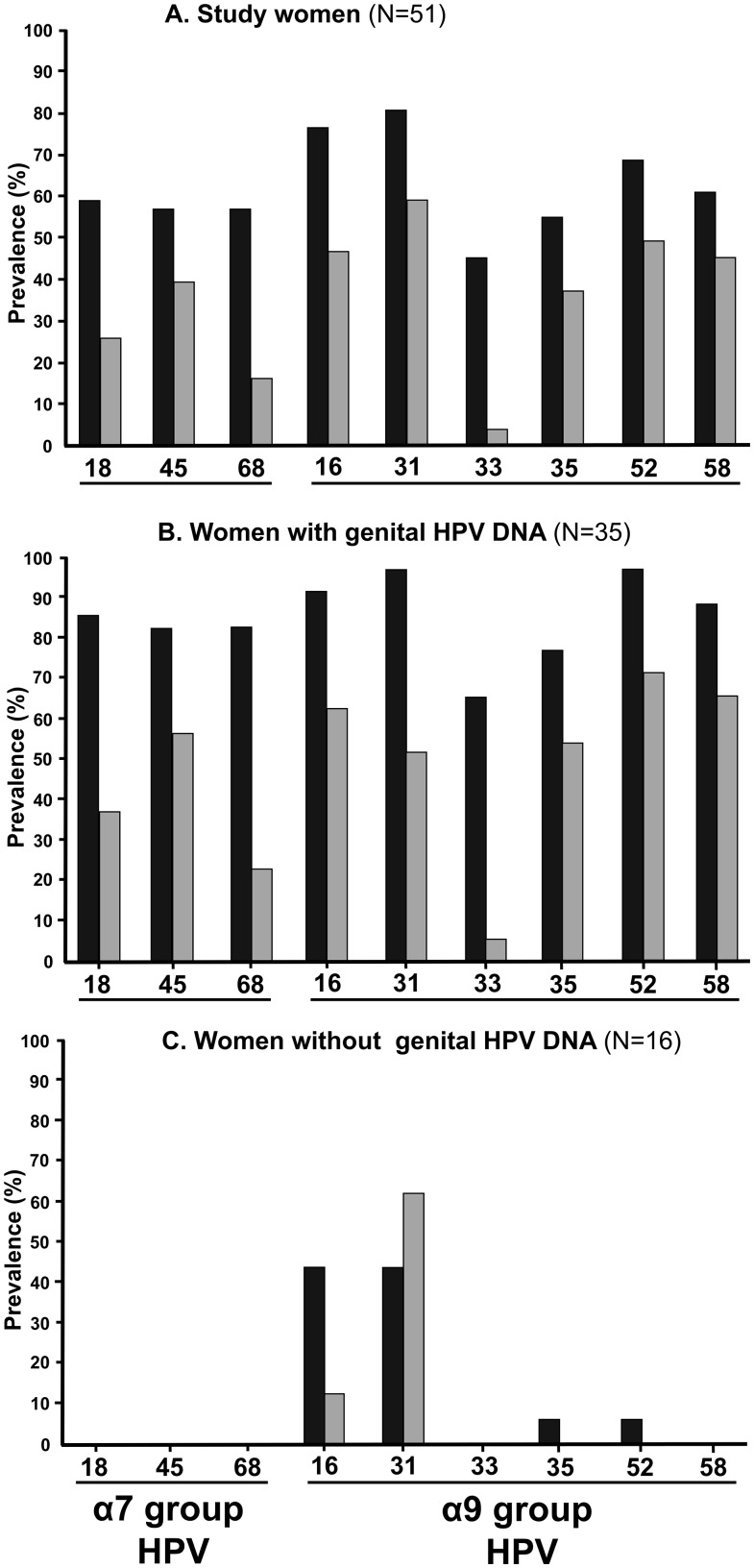
Prevalences of IgG reactivities to α7/α9 HPV types in paired serum (black boxes) and cervicovaginal secretions (grey boxes) according the presence of genital HPV DNA. **A.** Whole study population of first-generation immigrate African women (N = 51); **B.** Women with positive HPV DNA detection in their genital tract (group I; N = 35); **C.** Women with negative cervicovaginal HPV DNA detection (group II; N = 16).

In the systemic compartment, the large majority of women (90.2%; n = 46) exhibited serum IgG responses against at least one α7/α9 HR-HPV type, while only five women (9.8%) were seronegative for all the HPV types studied. Profiles of seropositivity to multiple HPV types were frequent (76.5%), on average against 7.1 VLP-HPV types (range, 2 to 9) per woman. Nearly half of women (43.1%) were seropositive for all the nine HPV types. The Fig 2 A depicts the α7 and α9 HPV-specific serum IgG responses of the study women. Overall, women were mostly seropositive for α9 HPV types, with serum IgG against HPV-31 (80.4%) being the most prevalent HPV-specific reactivity, followed by IgG to HPV-16 (76.5%), HPV-52 (68.6%) and HPV-58 (60.8%). The serum IgG to HPV-18 were detected in 58.8% of women, followed by IgG to HPV-68 (56.8%), HPV-45 (56.8%), HPV-35 (54.9%) and HPV-33 (45.1%). There was no significant difference in the IgG systemic reactivity between HIV-infected and uninfected women (Table 2).

**Fig 3:**
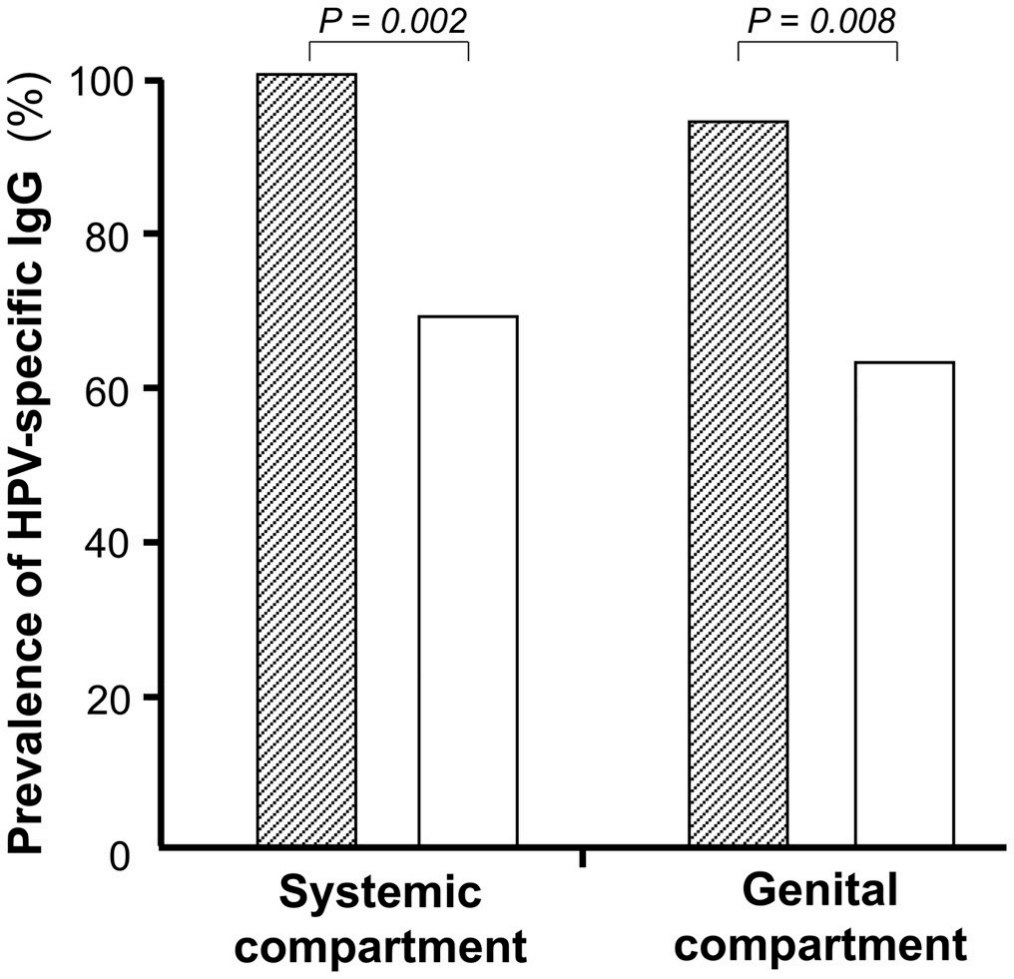
Prevalences of IgG reactivities to α7/α9 HPV types in paired serum and cervicovaginal secretions from study women with positive HPV DNA detection in their genital tract (group I; N = 35) (black hatched boxes), and from women with negative cervicovaginal HPV DNA detection (group II; N = 16) (white boxes). The prevalences of HPV-specific IgG reactivities by virus-like particles-based ELISA in the systemic as well as genital compartment were higher in women carrying HPV DNA than in women without detectable cervicovaginal HPV.

Serum HPV-specific IgG responses were more frequently detected in women with HPV genital (group I) than in women without genital HPV (group II) [group I: 100% (35/35) *versus* group II: 68.7% (11/16); *P = 0*.*002*] (Fig 3). Furthermore, the mean number of serum IgG reactivities to α7/α9 HR-HPV types were significantly higher in women from group I than in those from group II (group I: 7.7 HR-HPV types per woman, range: 1 to 9, *versus* group II: 1.0 HR-HPV type per woman, range: 0 to 2; *P<0*.*001*) (Fig 2B and 2C). Interestingly, while all (100%) women belonging to group I were seropositive against at least one HR-HPV type, one-third (31.3%, 5/16) of women from group II were seronegative for all HR-HPV investigated.

Women from group II harbored serum IgG only reactive to α9 HPV types, mostly to HPV-16 and HPV-31 (43.7%, 7/16) and less frequently to HPV-35 and HPV-52 (6.2%, 1/16) (Fig 2C). In addition, all women from group Ia, but one (ID#26), harbored concordant HPV-specific serum IgG immune responses for at least one of the HR-HPV types detected by PCR in their swab-collected cervicovaginal secretions (Table 3). Finally, the mean number of cross-reacting HPV-specific serum IgG reactivities against HPV types different from detected cervicovaginal HPV DNA was higher in women from group I than in those from group II (group I: 6.7 serum IgG reactivities per woman, range 1–8, *versus* group II: 1.0, range 0–2; *P<0*.*001*) (Table 3).

### Cervicovaginal HPV-specific IgG immune response

In the genital compartment, more than three-quarter (84.3%) of study women showed cervicovaginal IgG against at least one of the nine α7/α9 VLP-HR-HPV used as antigens, while no genital HPV-specific IgG could be detected in 15.7% (8/51) of women (Table 3 and Fig 2A). Genital IgG responses against several HR-HPV types were observed in more than half of study women (56.8%), with an average of 5.1 genital IgG reactivities to HR-HPV types (range, 2 to 9) per woman. Overall, women were mostly positive for α9 HPV types, with genital IgG against HPV-31 (58.8%) being the most prevalent HPV-specific reactivity, followed by IgG to HPV-52 (49.1%), HPV-16 (47.1%), and HPV-58 (45.1%). The other genital IgG were detected against HPV-45 (39.2%), HPV-35 (37.2%), HPV-18 (25.5%), HPV-68 (15.7%) and HPV-33 (3.9%). HIV-infected women were significantly more reactive for at least 6 HR-HPV in the cervical compartment than HIV-uninfected women (P < 0.05) (Table 2).

Similarly to the systemic compartment, genital HPV-specific IgG responses were more frequently detected in women with HPV genital shedding than in those without genital HPV [group I: 94.3% (33/35) *versus* group II: 62.5% (10/16); *P = 0*.*008*] (Fig 3). Furthermore, the mean number of genital IgG reactivities to α7/α9 HR-HPV types were significantly higher in women from group I than in those from group II (group I: 4.3 HR-HPV types per woman, range: 0 to 9, *versus* group II: 0.7 HR-HPV type per woman, range: 0 to 2; P<0.001) (Fig 2B and 2C). Interestingly, while all (100%) women belonging to group I were seropositive against at least one α7/α9 HR-HPV type, women from group II showed genital IgG only reactive against two α-9 HR-HPV types (HPV-16: 12.5% and HPV-31: 62.5%) (Fig 2B and 2C).

The majority of women from group I (54.3; 19/35) exhibited vaginal IgG response specific to the HR-HPV type detected in their cervicovaginal swab specimen (Table 3). However, 40.0% (14/35) of women from group I showed genital IgG response specific to other HR-HPV types than to the HPV type detected in their cervicovaginal secretions (Table 3). Finally, two (5.7%) women harboring genital DNA did not show any HPV-specific cervicovaginal IgG response.

Women from group II harbored genital IgG only reactive to α9 HPV types, including HPV-31 (62.5%, 10/16) and HPV-16 (12.5%, 2/16) (Fig 2C). The mean number of cross-reacting HPV-specific genital IgG reactivities against HPV types different from detected cervicovaginal HPV DNA was higher in women from group I than in those from group II (group I: 3.8 genital IgG reactivities per woman, range 0–9, *versus* group II: 0.7, range 0–2; *P<0*.*001*) (Table 3).

### Comparison of HPV-specific IgG immune responses in paired serum and cervicovaginal secretions

Overall, the distribution of serum HPV-specific IgG mirrored somehow that of genital HPV-specific IgG, with anti-VLP-HPV-31 (80.4% in serum and 58.8% in genital secretions), HPV-16 (76.5% in serum and 47.1% in genital secretions), HPV-52 (68.6% in serum and 49.1% in genital secretions) and HPV-58 (60.8% in serum and 45.1% in genital secretions) being the most frequently detected IgG antibodies both in the systemic and the genital compartments (Fig 2A).

There was however numerous differences between the systemic and genital compartments concerning the prevalences of IgG reactivities, the concordances between HPV-specific IgG reactivities and the types of detected genital HPV DNA, and the cross-reactivities of HPV-specific IgG against HPV types different from genital HPV DNA, mainly in the group I of women with HPV genital shedding (Fig 2B).

Thus, in the whole study population, the mean number of IgG reactivities to α7/α9 HR-HPV types was significantly higher in serum than in paired genital secretions (serum IgG: 5.6 HR-HPV types per woman, range 0 to 9, *versus* genital IgG: 3.2 HR-HPV types per woman, range 0 to 9; *P<0*.*001*) (Table 3). These observations were more pronounced in the group I of women shedding HPV DNA (serum IgG: 7.7 HR-HPV types per woman, range 1 to 9, *versus* genital IgG: 4.3 HR-HPV types per woman, range 0 to 9; *P<0*.*001*).

More than one-third (37.1%, 13/35) of women carrying genital HPV DNA (group I) did not show concordant IgG seroconversion profile against the ongoing HPV infection between the systemic and genital compartments (Table 3). Although these women showed serum IgG reactivities associated with HPV types detected from genital swab, they did not exhibited the same HPV-specific IgG reactivities in genital secretions. Furthermore, in the group Ia of women with genital HR-HPV DNA shedding, the concordance between HPV types detected by PCR and HPV-specific serum or cervicovaginal IgG reactivities appeared higher in the systemic than in the genital compartments. Indeed, while 26 out of the 27 (96.3%) women from the group Ia showed concordance between α7/α9 HR-HPV DNA and serum IgG to the same HR-HPV, only less than half of them (48.1%; 13/27) exhibited concordance between α7/α9 HR-HPV DNA, serum and genital IgG to the same HR-HPV (*P = 0*.*005*) (Table 3). Furthermore, a minority of women (7.4%; 2/27) with multiple genital HR-HPV DNA showed serum IgG responses against all the HR-HPV types detected in their genital swab, but their genital IgG responses were directed only against one of the HPV types detected by PCR (Table 3).

When excluding the IgG reactivities associated with HPV types detected from genital swab, the mean number of IgG cross-reactivities against HPV types different from genital HPV DNA was higher in serum than in paired genital secretions (serum IgG, 4.9 HR-HPV types per woman, range 0 to 8, *versus* genital IgG: 2.8 HR-HPV types per woman, range 0 to 8; *P<0*.*001*) (Table 3).

In contrast to group I, in women without genital HPV DNA shedding (group II), anti-VLP-HPV-31 was mostly detected in cervicovaginal secretions (62.5%) than in paired serum (43.7%) (Fig 2C). Nevertheless, in group II, the mean number of IgG cross-reactivities against HPV types different from genital HPV DNA was similar in serum and in paired genital secretions (serum IgG, 1.0 HR-HPV types per woman, range 0 to 2 *versus* genital IgG: 0.7 HR-HPV types per woman, range 0 to 2; *P > 0*.*05*) (Table 3).

HIV-infected and HIV-seronegative women harbored high rates of HR-HPV specific IgG positivity, in both serum (89.2% *versus* 92.8%) and genital secretions (86.5% *versus* 78.6%), without any significant difference (*P > 0*.*05*).

Finally, when considering both systemic and cervicovaginal compartments, 31.2% (5/16) of women without detectable genital HPV DNA (group II) did not show any serum and genital HPV-specific IgG immune responses to α7/α9 VLP-HR-HPV.

### Preliminary analysis on the presumed predictive efficiency of catch-up prophylactic Gardasil-9^®^ HPV vaccine in the study population

None of study women had received HPV prophylactic vaccination, which raises the issue of possible catch-up vaccination for them.

Study women could be divided in 3 distinct categories.

The first category comprises the majority of study women (68.6%; 35/51) with genital α7/α9-HR-HPV (group Ia) or other LR-HPV genotypes (group Ib). All these women harbored serum IgG reactivity profiles against an average of 6.1 Gardasil-9^®^ HR-HPV types per woman (range, 1 to 7), and genital IgG reactivity profiles against an average of 3.7 Gardasil-9^®^ HR-HPV types per woman (range, 0 to 7), likely indicating several past-history of genital infections by α7/α9 HR-HPV. Simultaneous IgG seropositivity to all HR-HPV types included in the nonavalent Gardasil-9^®^ vaccine was observed in 68.6% (24/35) of serum or genital secretions from women belonging to group I. However, partial simultaneous IgG seropositivity for 4 to 6 HR-HPV types included in the nonavalent Gardasil-9^®^ vaccine could be observed in 9 of 35 (25.7%) women. Thus, the majority (94.3%; 33/35) of this first category of women showed serum or cervicovaginal IgG positivities against all, or at least 4, of the seven HR-HPV types targeted by the Gardasil-9^®^ vaccine, and could be *a priori* poorly eligible for catch-up HPV vaccination.

The second category was the majority (68.7%, 11/16) of women from group II exhibiting serum IgG reactivity profiles against an average of 1.4 Gardasil-9^®^ HR-HPV type per woman (range: 0 to 2) and genital IgG reactivity profiles against an average of 1.0 Gardasil-9^®^ HR-HPV type per woman (range: 0 to 2) (Table 3 and Fig 2C). Simultaneous IgG seropositivity for 3 to 7 HR-HPV types included in the Gardasil-9^®^ vaccine could not be observed in these women. Such serum or genital HPV-specific IgG profiles could be a priori eligible for possible catch-up HPV vaccination.

Finally, the third category consisted in one-third (31.2%, 5/16) of study women without detectable genital HPV DNA (group II) who, furthermore, did not show any serum and genital HPV-specific IgG immune responses to all α7/α9 VLP-HR-HPV (Table 3 and Fig 2C). These women could be likely fully eligible for possible catch-up prophylactic HPV vaccination.

### Functional analyses of HPV-specific IgG from paired serum and cervicovaginal secretions of study women

The dilutions for PsV-35, -52 and -58 did not reached sufficient luminescence signal above the cell control for background luminescence, suggesting the failure of PsV production or only a very weak yield of production (not shown), and these PsV were therefore not included in the neutralization assay. Finally, the PBNA was carried out with PsV-16, -18, -31, -33, -45 and -68 in paired serum and cervicovaginal samples from 4 study women randomly selected in the group I for HPV IgG functional analyses.

**Fig 4:**
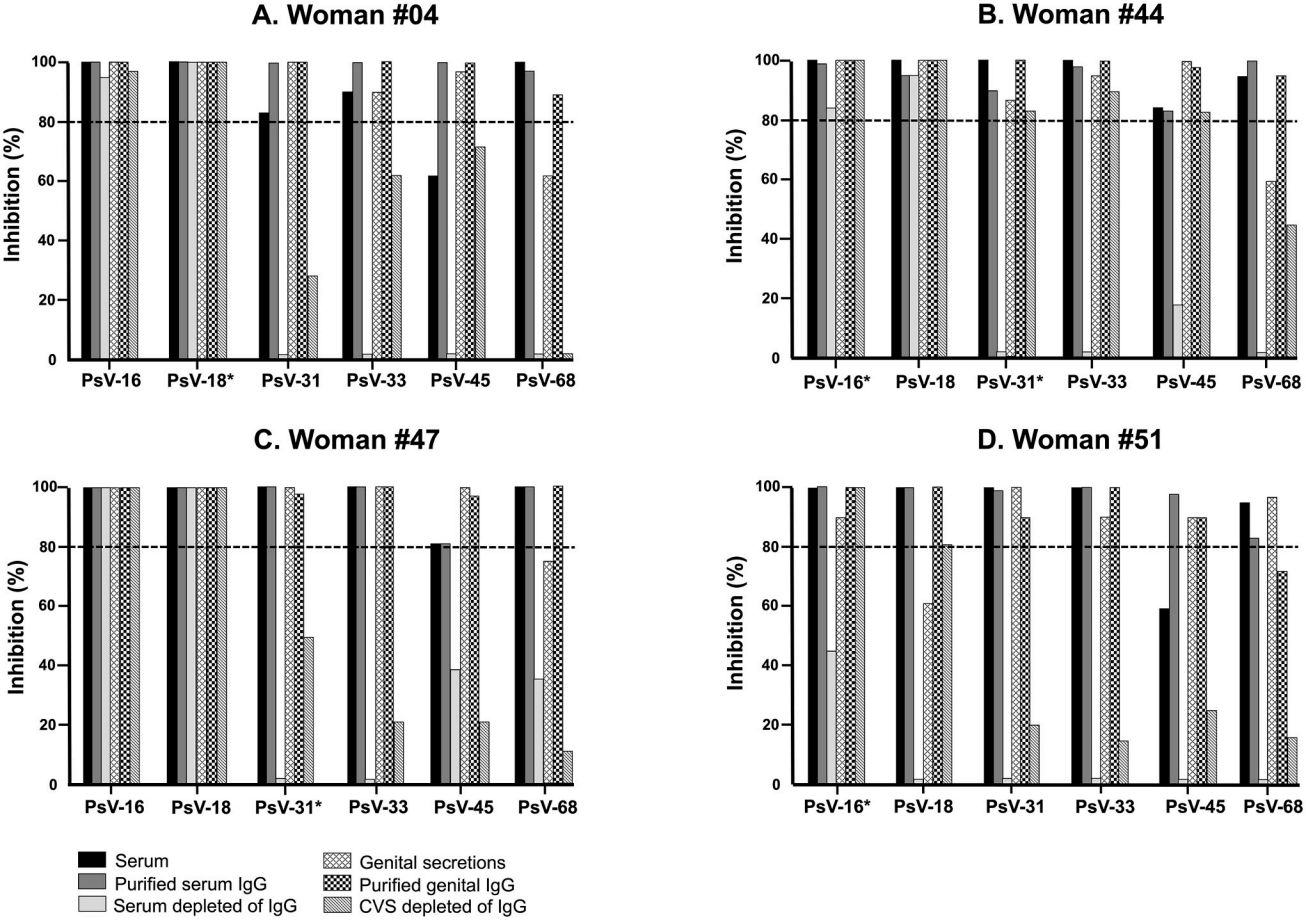
Inhibition of HPV PsV infection by purified IgG from serum and cervicovaginal washing of women infected with HPV. Results are displayed as the percentage of inhibition of infection by each HPV PsV in the presence of original serum (serum, black bar) and cervicovaginal secretions (CVS, bar with crossed dotted lines), purified serum IgG (serum IgG, dark grey bar) and genital IgG (IgG, bar with black tiles), and also serum depleted of IgG (light grey bar) and CVS depleted of IgG (bar with oblique lines) from woman #04 (**A**), #44 (**B**), #47 (**C**), and #51 (**D**). The hatched line represents the neutralization threshold. The star indicates the HPV genotype detected by PCR in vaginal swab.

The Fig 4 depicts the HPV PsV inhibition profiles and the in vitro neutralization capability of native serum and cervicovaginal secretions and their derived secondary samples from four selected study women. Overall, both purified serum IgG and purified genital IgG of all women induced more than 80% inhibition of the luminescent signal for all the PsVs types excepted for PsV-68 for which the purified genital IgG of the patient #51 did not succeed to neutralize the transduction of the COS-7 cells. Furthermore, all women showed both purified serum IgG and genital IgG with optimal neutralizing capacities (≥ 99%) against the PsVs homologous to the genotypes detected in their vaginal swab by PCR. When comparing the inhibition capacity between each native sample and its derived secondary samples, the inhibition capacity harbored by the native sample was quite similar to that observed for its purified IgG. While this inhibition capacity was dramatically reduced by half or totally disappeared for the sample depleted of IgG. Apart for the PsV-16 and PsV-18 for which the serum and CVS depleted of IgG of three women (#04, #44, #47) showed high neutralizing capacity (inhibition >80%) quite similar to both the native samples and the corresponding purified IgG. Depleted serum of woman #51 showed a total loss of its inhibition capacity for most of the PsVs studied, excepted for the PsV-16 for which the depleted serum harbored a residual inhibition capacity without neutralizing effect (Fig 4).

## Discussion

We herein addressed immunologically the issue of catch-up HPV vaccination in a series of immigrant African women living in France. Most of these women were infected with HIV and none of them had been vaccinated against HPV or undergone secondary prevention measures. Molecular detection of HPV DNA revealed unsuspected and atypical epidemiological profile of cervical HPV infection with high burden of HPV (68.6%) and α7/α9 HR-HPV (56.8%), the non-vaccine α7-HR-HPV-68 being the predominant genotype. In addition, serum and cervicovaginal IgG to at least one α7/α9 HR-HPV could be detected by VLP-based ELISA in the majority of included women, indicating a high frequency of past- or ongoing genital HPV infections. According to genital HPV detection and serum and genital IgG results, three distinct categories of women could be observed. Firstly, around two-third of women harboring genital HPV (group I) showed the remarkable association of the highest prevalences of both serum and cervicovaginal IgG responses, the highest mean number per woman of both serum and cervicovaginal IgG reactivities to α7/α9 HPV types and IgG reactivities to Gardasil-9^®^ HR-HPV types as well as the highest mean number per woman of both serum and cervicovaginal IgG cross-reactivities to α7/α9 HPV types different than those detected in genital secretions. In this category, concordant and cross-reacting IgG responses against α7/α9 HPV and Gardasil-9^®^ HR-HPV were highly prevalent in both the systemic and genital compartments. In addition, paired serum and cervicovaginal IgG from a subset of women of the group I showed large spectrum neutralizing capability against several α7/α9 HR-HPV-derived PsV. These results are likely evidence of significant past or ongoing exposure to genital HPV infection in these women and point that the reinforcement of the HPV-specific immune response by catch-up HPV vaccination in this category of women already naturally immune against Gardasil-9^®^ HR-HPV types will be hazardous. However, a catch-up HPV vaccination would be interesting to reinforce the natural humoral response already in place in these women by inducing vaccine-derived HPV-specific neutralizing IgG. Secondly, around one-fifth of study women had not genital HPV DNA (group II), and showed limited IgG response only against α9 HR-HPV targeted by the Gardasil-9^®^ vaccine (HPV-16, -31 and -52) in the systemic or genital compartments. These findings suggest limited past exposure to genital HPV, making likely the possibility of reinforcement of the HPV-specific humoral response by catch-up HPV vaccination. Thirdly, around one-tenth of study women were free of HPV DNA (group II), and did not show any IgG response to α7/α9 HPV, indicating a lack or only limited past exposure to genital HR-HPV. These features suggest, at least immunologically, the possibility to easily induce in this latter category of African immigrant a protective HPV-specific IgG response by catch-up HPV vaccination. Taken together, molecular detection of genital HPV DNA and assessment by VLP-based ELISA of IgG to α7/α9 HR-HPV in both systemic and genital compartments from first-generation immigrant African women living in France revealed in two-third of women the unexpected high burden of past or ongoing genital infection by frequent atypical HR-HPV types, likely reflecting the primary HPV epidemiology of contamination, and a strong and cross-reacting HPV-specific IgG immune response. However, around one-third of study women could, nevertheless, be eligible to catch-up HPV vaccination which could be beneficial regardless of their age, as they are still sexually exposed to the risk of contamination with oncogenic HPV.

In these African immigrant women, the prevalence of cervical HPV (68.6%), frequently associated with HR-HPV genotypes (52.9%), was particularly high. Such high prevalences of cervical HPV and HR-HPV appear uncommon and higher than HPV prevalences previously reported in the French female general population with cervical HR-HPV infection rates oscillating from 8.0% to 22.8% in HIV-negative adult women with normal cytology [23,65–67] and 26.4% in HIV-positive women [67]. A large European population-based survey on HIV-positive women reported cervical HR-HPV prevalences around 35.0% [68], much lower than that found in our study population. On the other hand, the high prevalences of HR-HPV infection found in the present study mirror the burden of cervical HR-HPV infection commonly reported in Sub-Saharan Africa. Indeed, quite similar to our study, cervical HR-HPV prevalences in sub-Saharan Africa can reach 46.2% in HIV-negative adult women (more than 25 years) and 79.1% in HIV-positive women [69–71]. These observations highlight that immigrant women originating from sub-Saharan Africa and living in France, irrespectively of their HIV-status, are at least twice more infected by HR-HPV than both the French and European female population. As a consequence, adult first-generation African immigrant women living in France, especially those infected with HIV, should be at very high-risk for developing cervical cancer.

Natural infection by HPV induce both local and systemic humoral response directed to HPV antigens testifying to the generalization of the humoral response beyond the site of virus intrusion [72]. In the present study, circulating IgG reactivities to at least one α7/α9 HR-HPV were detected in the majority (>90%) of women, independently of HIV infection. Furthermore, the prevalences of serum HPV-specific IgG were higher in women with genital HPV than in those without genital HPV, with a close concordance observed between the detected α7/α9 HR-HPV and the serum IgG reactivities. Interestingly, serum and genital IgG showed a large spectrum of neutralizing capability against several α7/α9 HR-HPV-derived PsV. The high seroprevalence rates of IgG to α7/α9 HR-HPV in study women are likely the witness of both past and ongoing genital exposition to HR-HPV in these African immigrant women living in France. Indeed, circulating HPV-specific IgG may persist longtime after the natural clearance of HPV infection by the host immune system and could therefore constitute a marker of past exposition to the virus [42,50,64,72]. Furthermore, the concordance between the serum IgG reactivity and HPV DNA detected indicates the activation of the humoral response against the ongoing HPV infection [48,64,72]. The relatively advanced age of our study women could have also been associated with particularly high rates of HPV-specific IgG seropositivity, as reported in previous studies showing increasing HPV-specific seropositivity with older age [73,74]. Thus, the high level of circulating IgG to α7/α9 HR-HPV types in study population could mirror the prolonged maintenance of HPV exposure and a subsequent reinforcement of the IgG response. Such high level of IgG responses likely indicate a truly increased cumulative incidence of HR-HPV infections both in HIV-negative and HIV-positive study women, and the lack of waning of HPV-specific antibodies contrarily to that generally occurred with age and the frequent decline of HPV exposure [75].

Similarly to circulating HPV-specific IgG, cervicovaginal IgG reactivities to at least one α7/α9 HR-HPV type were detected in the majority (>84%) of enrolled women, independently of HIV infection, and most particularly in women showing HPV DNA. The distribution profiles of genital HPV-specific IgG mirrored somehow that of serum HPV-specific IgG. However, the mean number of IgG reactivities to α7/α9 HR-HPV as well as the concordance between HPV types detected by PCR and HPV-specific serum or cervicovaginal IgG reactivities were higher in the systemic than in the genital compartments. Furthermore, less than half of women carrying genital HR-HPV DNA exhibited concordant serum and genital IgG responses against the α7/α9 HR-HPV detected in their genital secretions. Taken together, both systemic and genital humoral responses could not be considered as equivalent regarding the HPV-specific IgG response. Cervicovaginal HPV-specific monomeric IgG are thought to be mainly transudative from circulating IgG, as previously demonstrated by circulating vaccine induced IgG to HPV which transudate and/or exudate from the systemic compartment to the female genital mucosa to provide protection against HPV infections [76,77]. In addition, an active mucosal cervicovaginal secretion of HPV-specific IgG may be induced by HPV particles uptake from the genital mucosal and in fine by the development of cervical lesions [37,51,78,79]. In study women, HPV-specific genital IgG correlated only moderately with type-specific serum IgG responses, supporting the hypothesis that the genital mucosa is a poor inductor site for the immune response, with low rate of genital seroconversion and disappearance of genital IgG after the clearance of HPV infection [51,80]. In addition, other HPV infection sites could have elicited a systemic IgG response to HPV. Thus, because the rectal mucosa is a powerful inductor site [81,82], anal infection with α7/α9 HR-HPV could induce serum IgG against several α7/α9 HR-HPV, without detection of any α7/α9 HR-HPV DNA in genital swab.

Considering the facts that the development of HPV-induced seroconversion is not systematic after natural infection [42], that it often takes several months before seroconversion can be detected [42], and that most transient HPV infection do not elicit a detectable antibody response [42], Dillner and colleagues suggested that HPV-specific antibodies do not play an important role in the protection or clearance of HPV infections but only represent a serological scar or at most a marker of exposure [83]. However, although still debated [64], more recent studies and meta-analysis have highlighted some evidences about the modest protecting role against subsequent infection played by the naturally acquired HPV-type specific antibodies [72,84–87]. Note that in our series, serum or cervicovaginal antibodies to α7/α9 HR-HPV did not have protected women against an ongoing genital infection with phylogenetically closed genotypes of α7 or α9 HR-HPV species, likely indicating that partial protection conferred by natural HPV-specific IgG is basically HPV-type specific, as previously shown in unvaccinated, HPV-antibody-positive individuals [45,88,89].

The immunological basis of prophylactic HPV vaccination should take into account the existing serum or cervicovaginal antibodies to α7/α9 HR-HPV types targeted by Gardasil-9^®^ vaccine. The working hypothesis is that the presence of naturally derived IgG serum or cervicovaginal antibodies to α7/α9 HR-HPV types targeted by the Gardasil-9^®^ vaccine could, if being neutralizing antibodies, protect against the same type of HPV, as previously reported [72]. If one take into consideration that natural IgG serum or cervicovaginal antibodies to α7/α9 HR-HPV types protect only from reinfection, and cannot protect against HR-HPV phylogenetically closed to the same α7 or α9 group, the possibility exists that catch-up HPV vaccination could be interesting in this high-risk population whatever the level and cross-reactivity of its HPV-specific humoral responses in the systemic or genital compartments. Indeed, the HPV-specific antibody levels after vaccination in serum are 10–100 times higher as compared to naturally derived antibody levels, and the vaccine-derived specific antibodies show a significant 3 times higher avidity for VLP-HR-HPV than naturally acquired antibodies [45,90]. Thus, HPV vaccination could both reinforce the already existing HPV-specific humoral immunity and also induce new antibodies response against Gardasil-9^®^ α7/α9 HR-HPV types not yet encountered.

Otherwise, current prophylactic HPV vaccines are thought to confer better protection against HPV infection mainly in individuals who have not been yet infected [11,12]. Thus, one can consider eligible for catch-up HPV vaccination, at least theoretically, all women who have not been infected by all seven Gardasil-9^®^ vaccine α7/α9 HR-HPV types or yet infected by only a minority (1 to 3) of them. In the present series, Gardasil-9^®^ vaccine would be beneficial depending on the group of study women. Given that 96.5% (28/29) women showed serum or cervicovaginal IgG seropositivity against 4 to 7 HR-HPV types included in the Gardasil-9^®^ vaccine in group Ia, 16.7% (5/6) in group Ib, and 0% (0/16) in group II, catch-up HPV vaccination could be then potentially beneficial in around one-third (35.3%; 18/51) of study women who have not yet be infected by Gardasil-9^®^ HR-HPV types or infected by only 1 to 6 of them [91,92].

Taken together, catch-up HPV vaccination could be poorly feasible in the category of study women showing HR-HPV because of marked circulating or genital IgG to vaccine α7/α9 HR-HPV types, likely possible in the category of women without genital HPV DNA and with weak serum or cervicovaginal IgG to vaccine α7/α9 HR-HPV types, and fully efficient in the category of women without genital HPV DNA and without detectable serum and cervicovaginal IgG to α7/α9 HR-HPV types. Note that catch-up vaccination could be proposed even in HIV-infected women, in whom better immunogenicity is seen when HIV viral replication is controlled, and when there is no overt immunodeficiency [93,94]. For the majority of study women, especially those currently infected with HR-HPV, catch-up prophylactic HPV vaccination seems to be compromised. For this category of women at risk for cervical cancer, intensive HPV molecular testing, cytological screening for precancerous cervical lesions and adequate treatment, along with effective antiviral therapy of HPV, remain the best suitable preventive approach to avoid the occurrence of cervical cancer [33,34,95,96].

Our study findings should be interpreted in light of some limitations. Indeed, the study was constrained both by limited sample size of enrolled women, the only one center of inclusion, and its cross-sectional design. Thus, further studies with larger sample size are needed to provide more generalizable data in order to support our preliminary conclusions on the differential eligibility for the catch-up HPV vaccination in adult women living in France at-risk for HPV infection and related diseases. Furthermore, it should have been relevant to evaluate also the HPV-specific IgA immune response, since the natural and vaccine responses to HPV involve both IgA and IgG isotypes. Indeed, despite the depletion of IgG, cervicovaginal secretions of a subset of women with HR-HPV infection showed neutralizing capability against several α7 and α9 HR-HPV types, suggesting another source of neutralization in cervicovaginal secretions than HPV IgG antibodies. Such neutralizing capability of cervicovaginal secretions depleted of IgG would be attributable to mucosal HPV-specific IgA. Indeed, cervicovaginal HPV-specific s-IgA are only locally produced [36], are thought to reflect current genital HPV infection [37] or HPV-related cervical lesions [46], thus may reflect the acquired mucosal humoral immunity to HPV of the vaginal cavity more accurately than HPV-specific transudative IgG.

This study has also important strengths, including prospective recruitment and the evaluation of the humoral response in two body compartments, the systemic and genital compartments, which are not equivalent regarding HPV infection. It is the first study to evaluate the immunological basis of catch-up prophylactic HPV vaccination in the vulnerable population of African immigrants.

In conclusion, this study shows several unexpected immunological combinations of systemic and genital natural HPV-specific IgG responses and HPV genital shedding in first-generation immigrant African women living in France. The high HPV prevalence and the limited evidence that naturally acquired antibodies protect against new DNA detection, suggest that some first-generation immigrant African women could benefit from highly multivalent HPV vaccination. Around two-third of study women showed frequent ongoing cervical HPV infection and high rates of circulating and genital IgG against α7/α9 HPV, generally cross-reacting and with a large spectrum of neutralizing capability, avoiding, in practice, further possibility of catch-up vaccination. Nevertheless, about one-third of women had no evidence of previous HPV infection, or showed only low levels of genital and circulating IgG against α7/α9 HPV and could therefore be eligible for catch-up vaccination. On a public heath point of view, these observations rise the issue of possible interest to design new strategy of cervical cancer prevention, mostly in the direction of adult women at risk for HPV infection and associated lesions and who do not fit within the current WHO or national prophylactic HPV vaccine recommendations of prophylactic HPV vaccination at best prior the onset of sexual life. Instead of the age criterion, new strategy for catch-up HPV vaccination could include the results of the currently recommended primary molecular HPV testing in women aged from 30 to 65 years, and to a lesser extent assessment of the natural acquired humoral immunity to HPV (if serological tests for HPV infection will be available in routine). Studies, including mathematical modeling, assessing the efficacy and cost-effectiveness of catch-up multivalent HPV vaccine, are warranted in this population.

## Supporting information

S1 Data (XLSX)

S2 Data (DOCX)

S3 Data (DOCX)
