# Supplementary material for: Serum and cervicovaginal IgG immune responses against α7 and α9 HPV in non-vaccinated women at risk for cervical cancer: Implication for catch-up prophylactic HPV vaccination
Source: PLoS One. 2020 May 18;15(5):e0233084. doi: 10.1371/journal.pone.0233084 (PMC7233543; doi:10.1371/journal.pone.0233084)
Supplement: S2 Data — (DOCX) [file pone.0233084.s002.docx]

**QUESTIONNAIRE DE L’ETUDE (Page 1)**

N°: Etiquette:

Age (ans ; mois):

Immigration^*^ (génération): **1^ère^** **2^ème^** **3^ème^**

Pays de naissance (**si 1^ère^ génération**):

Durée de résidence en France (**si 1^ère^ génération**):

Statut VIH connu: **OUI** **NON**

Site d'inclusion: **MIT**  **Gynécologie**

Critère d'inclusion (notamment, respect du délai d'abstinence sexuel de 3 jours avant les prélèvements):

**OUI NON**

Lésions associées aux VPH (**service de gynécologie**):

**Inconnue ASCUS CIN1**

**CIN2 CIN 3 Cancer**

Statut Marital: **Célibataire** **en couple Mariée** **Divorcée** **Veuve**

Occupation professionnelle: **Etudiante Sans emploi Employée**

Niveau d'étude: **Aucun niveau d'étude Etudes primaires Lycée Etudes universitaires**

Age 1^er^ rapport sexuel:

Partenaire régulier: **OUI NON**

Multipartenariat^**^: **OUI NON**

Si **OUI** combien:

Avoir entretenu des rapports sexuels contre rémunération:

**OUI NON**

Si **OUI** à quelle fréquence: **Régulièrement Occasionnellement**

Fumeur (tabagisme): **OUI NON**

**QUESTIONNAIRE DE L’ETUDE (page 2)**

Infection sexuellement transmissible (IST) au moment du prélèvement: **OUI NON**

Si **OUI** la ou les quelle(s):

Antécédents d’IST: **OUI NON**

Si **OUI** la ou les quelle(s):

Antécédents d'infection à *Herpès simplex virus*: **OUI NON**

* 1^ère^ génération: personne née étrangère à l’étranger, et résidant en France (domicile principal); 2^ème^ génération: personne née en France ayant au moins un parent

immigré; 3^ème^ génération: personne née en France ayant au moins un grand parent immigré [INSEE 1; INSEE 3].

**Rapports sexuels avec plus d'un partenaire du sexe opposé au cours des douze mois précédant l'enquête [Bajos et *al*., 1991].
