# Supplementary material for: Serum and cervicovaginal IgG immune responses against α7 and α9 HPV in non-vaccinated women at risk for cervical cancer: Implication for catch-up prophylactic HPV vaccination
Source: PLoS One. 2020 May 18;15(5):e0233084. doi: 10.1371/journal.pone.0233084 (PMC7233543; doi:10.1371/journal.pone.0233084)
Supplement: S3 Data — (DOCX) [file pone.0233084.s003.docx]

**STUDY QUESTIONNAIRE (Page 1)**

N°: Label:

Age (years ; month):

Immigration (generation): **1^st^** **2^nd^** **3^rd^**

Country of birth (**if 1^st^ generation**):

Time of stay in France (**if 1^st^ generation**):

HIV statu: **YES** **NO**

Meet the inclusion criteria (respect of the period of sexual abstinence of 3 days before the samples):

**YES NO**

HPV-associated lesions :

**unknown ASCUS CIN1**

**CIN2 CIN 3 Cancer**

Marital status: **Single** **In life couple Married** **Divorced** **Widow**

Employment status: **Student Unemployed Employed**

Education level: **Never studied Elementary Highschool University**

Age at 1^st^ intercourse:

Regular sex partner: **YES NO**

Several sex partners: **YES NO**

If **YES** how much:

Having had intercourse for remuneration:

**YES NO**

If **YES** how often: **Regularly Occasionally**

Smoking: **YES NO**

**STUDY QUESTIONNAIRE (page 2)**

Sexually transmitted infection (STI) at the time of collection: **YES NO**

If **YES** which one (s):

History of STIs: **YES NO**

If **YES** which one (s):

History of genital herpes (HSV-2) : **YES NO**
